# Frequency of *APOE, MTHFR* and *ACE* polymorphisms in the Zambian population

**DOI:** 10.1186/1756-0500-7-194

**Published:** 2014-03-28

**Authors:** Masharip Atadzhanov, Mwila H Mwaba, Patrice N Mukomena, Shabir Lakhi, Peter Mwaba, Sruti Rayaprolu, James F Meschia, Owen A Ross

**Affiliations:** 1 Department of Internal Medicine, University of Zambia, P.O.Box 51237, Lusaka, Zambia; 2 Department of Neuroscience, Mayo Clinic, Jacksonville, FL 32225, USA; 3 Department of Neurology, Mayo Clinic, Jacksonville, FL 32225, USA

**Keywords:** Sub-Saharan Africa, Zambia, Frequency, *APOE*, *MTHFR*, *ACE* polymorphisms

## Abstract

**Background:**

Polymorphisms within the apolipoprotein-E (*APOE*), Methylenetetrahydrofolate reductase (*MTHFR*) and Angiotensin I-converting enzyme (*ACE*) genes has been associated with cardiovascular and cerebrovascular disorders, Alzheimer’s disease and other complex diseases in various populations. The aim of the study was to analyze the allelic and genotypic frequencies of *APOE*, *MTHFR* C677T and *ACE* I/D gene polymorphisms in the Zambian population.

**Results:**

The allele frequencies of *APOE* polymorphism in the Zambian populations were 13.8%, 59.5% and 26.7% for the ε2, ε3 and ε4 alleles respectively. *MTHFR* C677T and *ACE* I/D allele frequencies were 8.6% and 13.8% for the T and D minor alleles respectively. The ε2ε2 genotype and TT genotype were absent in the Zambian population. The genetic distances between Zambian and other African and non-African major populations revealed an independent variability of these polymorphisms.

**Conclusion:**

We found that the *APOE* ε3 allele and the I allele of the *ACE* were significantly high in our study population while there were low frequencies observed for the *MTHFR* 677 T and *ACE* D alleles. Our analysis of the *APOE, MTHFR* and *ACE* polymorphisms may provide valuable insight into the understanding of the disease risk in the Zambian population.

## Background

The burden of non-communicable diseases such as stroke and cardiovascular disease is increasing in Sub-Saharan Africa (SSA) [1,2]. Their development is most likely caused by the interaction of multiple genetic factors and known risk factors (hypertension, diabetes, dyslipidemia, and smoking). One of the challenges for translating disease associated polymorphisms into clinical application is the lack of knowledge regarding the frequency of the polymorphism in the targeted population. Without this information, population-attributable risk remains unknown. In addition, factors that could affect the association of the allele with disease, either positively or negatively, such as ethnicity and gender, may not be possible to determine without population based allele frequencies [3].

A number of genetic polymorphisms, such as Apolipoprotein E (*APOE*). Methylenetetrahydrofolate reductase (*MTHFR*) and Angiotensin I-converting enzyme (*ACE*), have been studied in relation to age-related disorders. Specific polymorphisms in these genes have been implicated in various complex disorders including cerebrovascular disease (CVD), coronary artery disease (CAD) and Alzheimer’s disease (AD) [4-7].

For example, ACE is an enzyme of the renin-angiotensin system (RAS) catalyzing the conversion of angiotensin I to angiotensin II which is implicated in fluid and electrolytes balance. Angiotensin II has vasoconstrictor effect hence contributing to systemic blood pressure control, it also inhibits the release of acetylcholine (anticholinergic effect) and has pro-inflammatory effect [8].

The *ACE* polymorphism is characterized by the presence (insertion or I) or the absence (deletion or D) of Alu Ya5 inside intron 16 giving three possible genotypes (homozygote **II,** heterozygote **ID** and homozygote **DD**). The frequency of the insertion/deletion I/D polymorphism of the *ACE* gene has been widely investigated since it was identified by Rigat et al. [8]. The authors further observed that the highest serum ACE activity was in the DD genotype as opposed to II genotype in which the lowest activity was found [9]. Many authors have suggested the *ACE* I/D variants predisposes the individual to CAD, hypertension, stroke and diabetes mellitus [10-17]. Several studies have reported a reduction of the incidence and rate of the cognitive decline in AD after starting treatment with RAS-acting antihypertensive drugs (ACE inhibitors) and angiotensin receptor blockers (ARB) [18].

The *APOE* gene encodes a protein which is essential for the normal catabolism of triglyceride rich lipoprotein constituents and modulates lipoprotein metabolism. The *APOE* gene is polymorphic with three common alleles, ε2, ε3 and ε4 resulting in three distinct protein isoforms Ε2, Ε3 and Ε4 determined by the two amino acid substitutions (R112C and C158R). Several investigations have suggested that the *APOΕ4* is the ancestral allele [19,20] even though it is the risk allele in many diseases such CAD [4], CVD [21] and AD [22]. MTHFR is a folate related enzyme important for remethylation of homocysteine to methionine. Elevated total plasma homocysteine (t-Hcy) concentration was found to be correlated with *MTHFR* C677T polymorphism. A common polymorphism of the *MTHFR* gene, C677T, has been reported to be associated with reduced enzyme activity and increased t-Hcy levels [23] and hence, an independent risk factor for stroke, CAD, and AD [24]. The allelic frequencies of these specific polymorphisms in the *APOE*, *MTHFR* and *ACE* genes substantially varies in different regions of the world and among ethnic groups [3,19,25,26] and have not yet been studied in the Zambian population, hence, association of them with stroke, CAD, AD, and other common non-communicable diseases in this population is unclear.

Zambia, officially the Republic of Zambia, is a landlocked country in the central part of southern Africa. Zambia covers an area of 752,614 square kilometers (290,586 square miles) and has a population of almost 13 million, giving the country one of the lowest populations-to-land ratios in Africa. It borders the Democratic Republic of the Congo to the north, Tanzania on the northeast, Malawi on the east, Mozambique, Zimbabwe, Botswana, and Namibia to the south, and Angola on the west [27]. The original inhabitants of all of modern day Zambia, except Western Province, are called Batwa (Khoisan). They were hunters and gatherers who lived a nomadic life. The Khoisans were the only inhabitants of most of Zambia until the 4th century, when they were displaced by the Bantu who started to migrate from the north.Between the 15th century (or possibly earlier) and the 18th century, various groups of Bantu migrants from the southern Congo settled in Zambia [28]. Zambia was influenced by two “invasions” in the mid-19th century. Shaka’s Zulu empire in South Africa set in motion a series of migrations, commonly referred to as the mfecane; groups of peoples, including the Ngoni. The other invasion came in the form of traders from the north. The territory of the present Zambia, being far inland, did not have direct contact with non-Africans until relatively recently in its history. The traders from the north (Nyamwezi, Arabs, and Swahili) drew Zambia into long-distance trading systems [28]. At present Zambia has a mixture of 72 ethnic tribes. However, there are seven (Chewa, Bemba, Lunda, Lozi, Kaonde, Luvale, and Tonga) major ethnic groups who belong to the Bantu people. The Tonga people were one of the first cultures to settle in Zambia. Industrialization and urbanization has seen these ethnically different people brought together by economic interests.

In this study we have evaluated the prevalence of *APOE*, *MTHFR*, and *ACE* gene polymorphisms in the Zambian population and compared those frequencies with African and other populations to provide baseline epidemiological data for future clinical investigations of CVD, CAD, AD, and other diseases in Zambia.

## Methods

### Sample collection

Blood samples were obtained from one hundred and sixteen (68 women, 48 men) unrelated subjects. The study included subjects between 25 and 60 years (mean age 44 ± 16 years), who indicated their ancestry and belonged to one of 72 ethnic Zambian tribes. All individuals were recruited as part of Zambian Stroke Study [29,30]. Available clinical records were analyzed for exclusion of major neurological diseases. Subjects known to have risk factors for stroke (hypertension, severe coronary artery disease, diabetes mellitus and hyperlipidemia) were excluded from the study. Pregnant women and HIV positive subjects were also excluded from the study. All participants gave written informed consent and the study was approved by the Biomedical Research Ethics Committee of the University of Zambia.

### Genetic analysis

Whole blood was collected in 4 ml EDTA tubes and stored at 4 degrees Celsius prior to DNA extraction. The DNA extraction was performed using silica-gel-based membrane with vacuum technology (QIAamp DNA Mini Kit (250) (QIAgen, London, UK).

Genotyping was performed using fluorescent-labeled PCR technology with ABI TaqMan probes and chemistry (Applied Biosystems, Foster City, CA), amplification was performed with an ABI7900 Real-Time PCR system and analysis performed using SDS 2.2.2 software. Positive and negative controls where included on all TaqMan assay plates. Positive or ambiguous results in the TaqMan assay were also confirmed / resolved with direct sequencing.

#### Statistical analysis

We determined genetic distance using Carvalli-Sforza and Edwards (1967) method [31] in order to give an overview of the relationship between the Zambian and other populations. The chi-square test was used to test whether observed allele frequencies agreed with those expected in the Hardy-Weinberg equilibrium (HWE). Differences with other populations were assessed using chi-square contingency test.

## Results

Genotype and allele frequencies for the Zambian population were as shown in Table 1. Genotyping call-rate was greater than 98% with less than 2% of samples requiring sequencing to resolve or confirm the genotype data. The distribution of *MTHFR* and *APOE* genotypes conformed to Hardy-Weinberg equilibrium (HWE) with *p = 0.540* and *p = 0.456* respectively, while the ACE allele distribution was not according to HWE (*p =* 0.005) due to an increase in rare minor allele homozygotes. There was no statistically significant difference between male and female data for *APOE*, *MTHFR* and *ACE* polymorphisms (Table 2). Our analysis of the *APOE* gene showed that the rare E2E2 genotype was absent in our sample. In addition, there was no significant difference between the frequency of the ε3ε3 and ε3ε4 genotypes (*p* > 0.999) which were the most frequent genotypes observed. The Ε3 allele of *APOE* polymorphism was found to be the most frequent (0.595), while Ε2 allele had a frequency of 0.138, and Ε4 0.267.

**Table 1:** **Allele and genotype frequencies of the *APOE*, *MTHFR *** **and *ACE *** **polymorphisms and HWE in the Zambian population (n = 116)**

**Polymorphism**	**Allele**	**Frequency**	**Genotype**	**% observed**	**% expected**	**HWE**
APOE						*p = 0.456*
	Ε2	0.14	Ε2Ε2	0	1.9	X2 = 4.684
	Ε3	0.6	Ε3Ε3	32.8	35.4	
	Ε4	0.27	Ε4Ε4	7.8	7.1	
			Ε2Ε3	21.6	16.4	
			Ε2Ε4	6	7.4	
			Ε3Ε4	31.9	31.8	
MTHFR C677T						*p = 0.540*
	C	0.91	CC	82.8	83.5	X2 = 1.233
	T	0.09	CT	17.2	15.7	
			TT	0	0.74	
ACE D/I						*p = 0.005*
	D	0.14	DD	5	1.9	X2 = 10.472
	I	0.86	ID	17.2	23.8	
			II	77.6	74.3	

**Table 2:** **Gender frequencies of the *APOE*, *MTHFR *** **and *ACE *** **polymorphisms in the Zambian population**

**Polymorphism**	**Allelic frequencies**				**Genotypic frequencies**			
		**m = 102**	**f = 130**	***p***		**m = 51**	**f = 65**	***p***
APOE	Ε2	0.137	0.138	0.57	Ε2Ε2	0	0	
	Ε3	0.598	0.592	0.52	Ε3Ε3	0.314	0.34	0.47
	Ε4	0.265	0.269	0.53	Ε4Ε4	0.078	0.08	0.62
					Ε2Ε3	0.235	0.2	0.41
					Ε2Ε4	0.039	0.08	0.33
					Ε3Ε4	0.333	0.31	0.46
MTHFR C677T								
	C	0.902	0.923	0.37	CC	0.804	0.85	0.36
	T	0.098	0.077	0.37	CT	0.196	0.15	0.36
					TT	0	0	
ACE D/I								
	D	0.127	0.146	0.42	DD	0.039	0.06	0.46
	I	0.873	0.854	0.42	ID	0.176	0.17	0.55
					II	0.784	0.77	0.51

**Figure 1:**
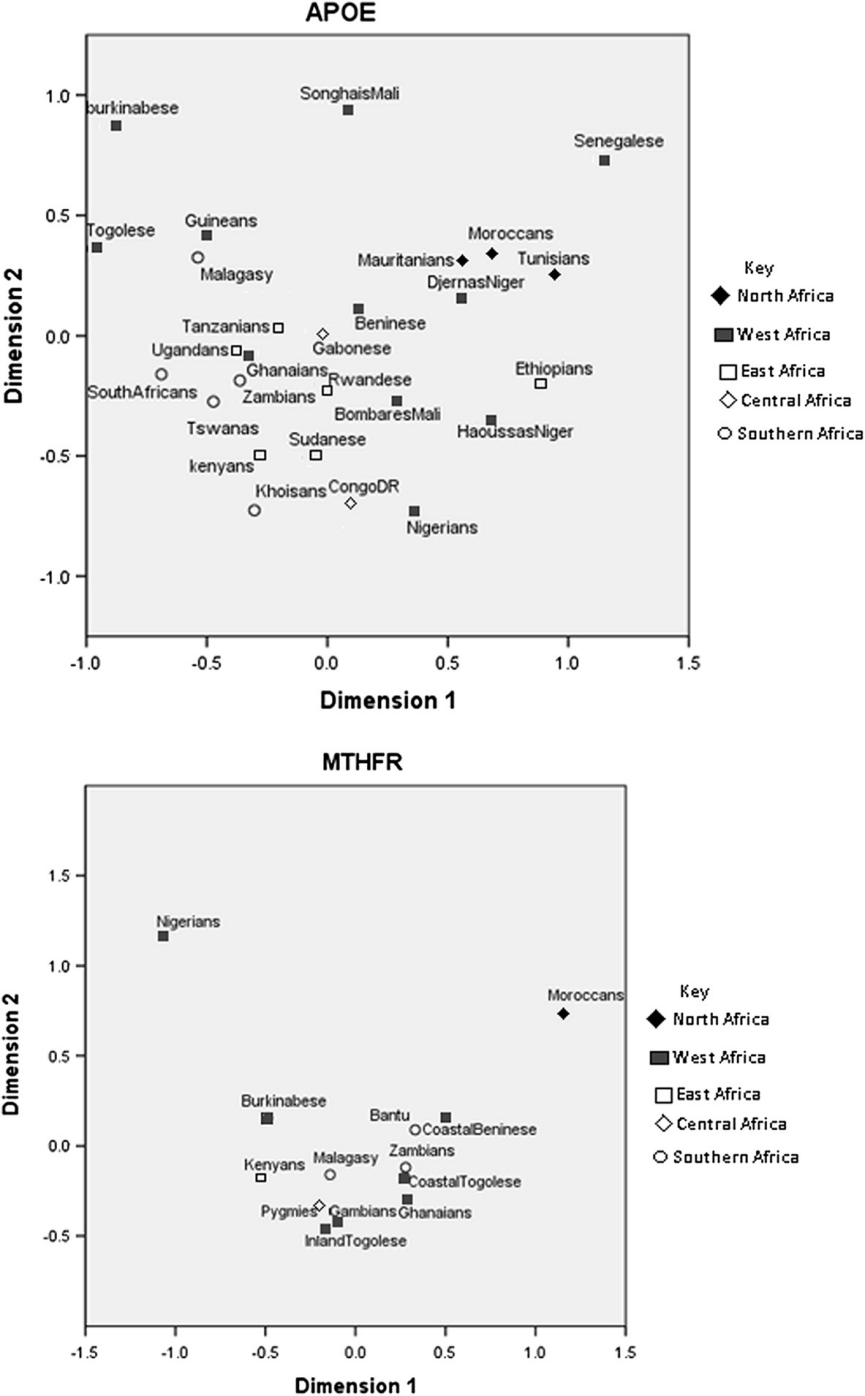
Genetic relationships between populations represented by Multidimensional Scaling Analysis for APOE and MTHFR.

The distribution of the *APOE* allele frequencies in the Zambian population has an intermediate position among other African populations. As shown in Table 3 and Figure 1, pooled *APOE* allele frequencies of the Zambian population was close to Tswanas [32], Ugandans [33], Ghanaians [32], and Tanzanians [34].

**Table 3:** **The *APOE *** **allele frequencies in the African populations**

	**n**	**ε2**	**ε3**	**ε4**	***p***
**North Africa**					
Mauritanians [35]	20	0.08	0.83	0.1	0
Moroccans [36]	100	0.07	0.85	0.09	0
Tunisians [37]	180	0.04	0.87	0.09	0
**West Africa**					
Ghanaians [32]	110	0.15	0.61	0.24	0.89
Gabonese [35]	25	0.12	0.68	0.2	0.48
Nigerians [38]	365	0.03	0.68	0.3	0.03
Bombareans (Mali) [35]	16	0.06	0.72	0.22	0.12
Guineans [35]	30	0.23	0.6	0.17	0.1
Beninese [19]	97	0.1	0.74	0.16	0.11
Togolese [35]	19	0.32	0.47	0.21	0.01
Djernas (Niger) [35]	16	0.06	0.81	0.13	0.01
Haoussas (Niger) [35]	37	0.03	0.78	0.19	0.01
Burkinabese [35]	20	0.38	0.5	0.13	0
Songhais (Mali) [35]	17	0.21	0.74	0.06	0
Senegalese [35]	33	0.03	0.94	0.03	<0.0001
**East Africa**					
Ethiopians [19]	164	0.03	0.81	0.14	0
Ugandans [33]	140	0.16	0.59	0.25	0.85
Tanzanians [34]	143	0.14	0.65	0.21	0.66
Rwandese [35]	21	0.1	0.67	0.24	0.62
Kenyans [34]	61	0.09	0.59	0.32	0.5
**Southern Africa**					
Zambians (current study)	116	0.14	0.6	0.27	current study
Tswanas [32]	100	0.15	0.57	0.29	0.86
South Africans [2]	505	0.190	0.52	0.29	0.43
Sudanese [35]	103	0.08	0.62	0.29	0.49
Khoisans [39]	247	0.08	0.55	0.37	0.2
Malagasy [35]	22	0.23	0.59	0.18	0.12
**Central Africa**					
Central Africa Republicans (Pygmies) [35]	70	0.06	0.54	0.41	0.05
Congolese (Congo DR) [35]	24	0.04	0.63	0.33	0.06
**Other**					
Sub-Saharans(SSA) [35]	470	0.12	0.71	0.18	0.27

The C allele of the *MTHFR* C677T polymorphism was higher than T allele (*p* < 0.0001) with frequency of 0.914 and 0.086 respectively. The TT genotype was absent in the Zambian population.

Multidimensional Scaling (MDS) analysis of the *MTHFR* C677T polymorphism in the African populations, including the Zambian population (Figure 1, Table 4) showed a close relationship with previous studies on the Bantu populations [40].

**Table 4:** **The *MTHFR *** **C677T polymorphism allele frequencies in the African populations**

	**n**	**C**	**T**	**p**
**North Africa**				
Moroccans [36]	182	0.73	0.27	<0.0001
**West Africa**				
Gambians [40]	24	0.94	0.06	0.69
Nigerians [41]	25	0.94	0.06	1
Burkinabes [42]	382	0.95	0.06	0.18
Coastal Togolese [43]	127	0.92	0.08	1
Inland Togolese [43]	68	0.94	0.06	0.58
Coastal Beninese [43]	270	0.9	0.1	0.85
Ghanaians [44]	174	0.92	0.08	1
**East Africa**				
Kenyans [40]	61	0.95	0.05	0.55
**South Africa**				
Zambians	116	0.91	0.09	Current study
Malagasy [40]	97	0.93	0.07	0.61
**Central Africa**				
Pygmies [40]	8	0.94	0.06	1
**Other**				
Bantu [40]	44	0.91	0.09	1
SSA [40]	234	0.93	0.07	0.51

The current study found that, based on *MTHFR* C677T polymorphism, Zambian population was closer to coastal Togo and Moroccans were found to be furthest from Zambians (Figure 1). Our data is consistent with published data which shows that the frequency of homozygous mutated genotype TT was absent in most of the investigated African populations [45] According to Murry et al. [46], this data suggests the lethal nature of this genotype in most SSA populations.

The genotypic data for *ACE* shows that the II genotype was higher than the ID genotype (*p* < 0.0001) and the DD genotype (*p* < 0.0001) in the Zambian population. The *ACE* polymorphism displayed I and D allele frequencies at 0.862 and 0.138 respectively.

The data shows that *APO ε3*, *MTHFR* 677C and *ACE* I alleles were most widespread in the Zambian population. Extensive interethnic variations in the frequency of *I* and *D* alleles have been reported worldwide for various populations (Table 5).

**Table 5:** **Allele frequencies of the *ACE *** **gene in different ethnic groups**

		**ACE allele frequency**		
	**n**	***I***	***D***	***P***
**North Africa**				
Moroccans [36]	182	0.23	0.77	<0.0001
Egyptians [47]	188	0.28	0.72	<0.0001
Algerians [15]	48	0.27	0.73	<0.0001
Tunisians [15]	47	0.24	0.76	<0.0001
**West African**				
Nigerians [48]	80	0.41	0.59	<0.0001
**East Africa**				
Sudanese [49]	121	0.36	0.64	<0.0001
Somalis [49]	53	0.27	0.73	<0.0001
**South Africa**				
Zambians	116	0.86	0.14	Current study
**Other**				
Omanis [49]	124	0.29	0.71	<0.0001
African Americans [15]	40	0.3	0.7	<0.0001
Emiratis [50]	159	0.34	0.66	<0.0001
Greeks [51]	84	0.42	0.58	<0.0001
Brazilians [52]	65	0.42	0.58	<0.0001
Chinese, Dahur [53]	84	0.43	0.57	<0.0001
Caucasians [54]	733	0.54	0.46	<0.0001
Indians [55]	166	0.54	0.46	<0.0001
Japanese [56]	136	0.65	0.35	<0.0001
Chinese [57]	189	0.71	0.29	0
Pima Indians [58]	305	0.71	0.29	0
Mexican Teeneks [59]	64	0.78	0.22	0.21
Yanomami Indians [48]	49	0.85	0.15	1
Samoans [48]	58	0.91	0.09	0.46
Australian Aborigines [60]	184	0.97	0.03	0
Abazians [46]	126	0	1	<0.0001

## Discussion

In this study we have analyzed for the first time polymorphisms of the *APOE*, *MTHFR* and *ACE* genes in the Zambian population. This constitutes an approach to study genetic factors considered as risk factors for CVD, CAD, and AD in this population.

In our study population the *APOE* allele frequencies of ε2 (0.138), ε3 (0.595), and ε4 (0.267) were within the reported range of African populations [19] and the ε3 was the most prevalent. These allele frequencies varied significantly between Zambian and the North African populations such as Moroccans [36] and Tunisians [37]. The variation may be explained by extensive intermixing of the North African populations with Arab, Jewish and other Western Eurasian populations.

The *APOE* ϵ2 allele frequency range between the Zambian and other African populations was from 7% (Tswana, Botswana) to 11.1% in Haoussa’s of Niger. This variation may be explained according to the ethnolinguistic classification of the African population which identifies the Haoussas as belonging to the Afro-asiatic macro-family while the Zambian population can be classified within the Niger-Kordofanian, the largest of the four ethnolinguistic African macro-families [61]. The *APOE ε3* allele frequency range between the Zambian population and other African populations was from 4% (Malagasy [35]) to 34.4% (in Senegalese [35]). The variation of *APOE ε4* allele frequency between the Zambian and other African populations was as low as 1.8% between Zambian and Tswana [32] populations and as high as 23.7% between the Zambian and Senegalese populations.

Patterns of genetic variation in modern African populations are shaped by demographic forces such as the Bantu migration [62]. Bantu speakers from West Africa practicing agricultural subsistence farming migrated throughout SSA and subsequently admixed with indigenous populations [61]. According to Gerdes et al. [63], populations with long-established agricultural economy such as the West African, [43,64] have higher ε3 allele frequency compared to other populations. Higher frequencies of *APOE* ε3 allele were found in populations around the Middle East probably because they have a longer established agricultural economy compared to West Africa, with the exception of Senegal [19].

**Figure 2:**
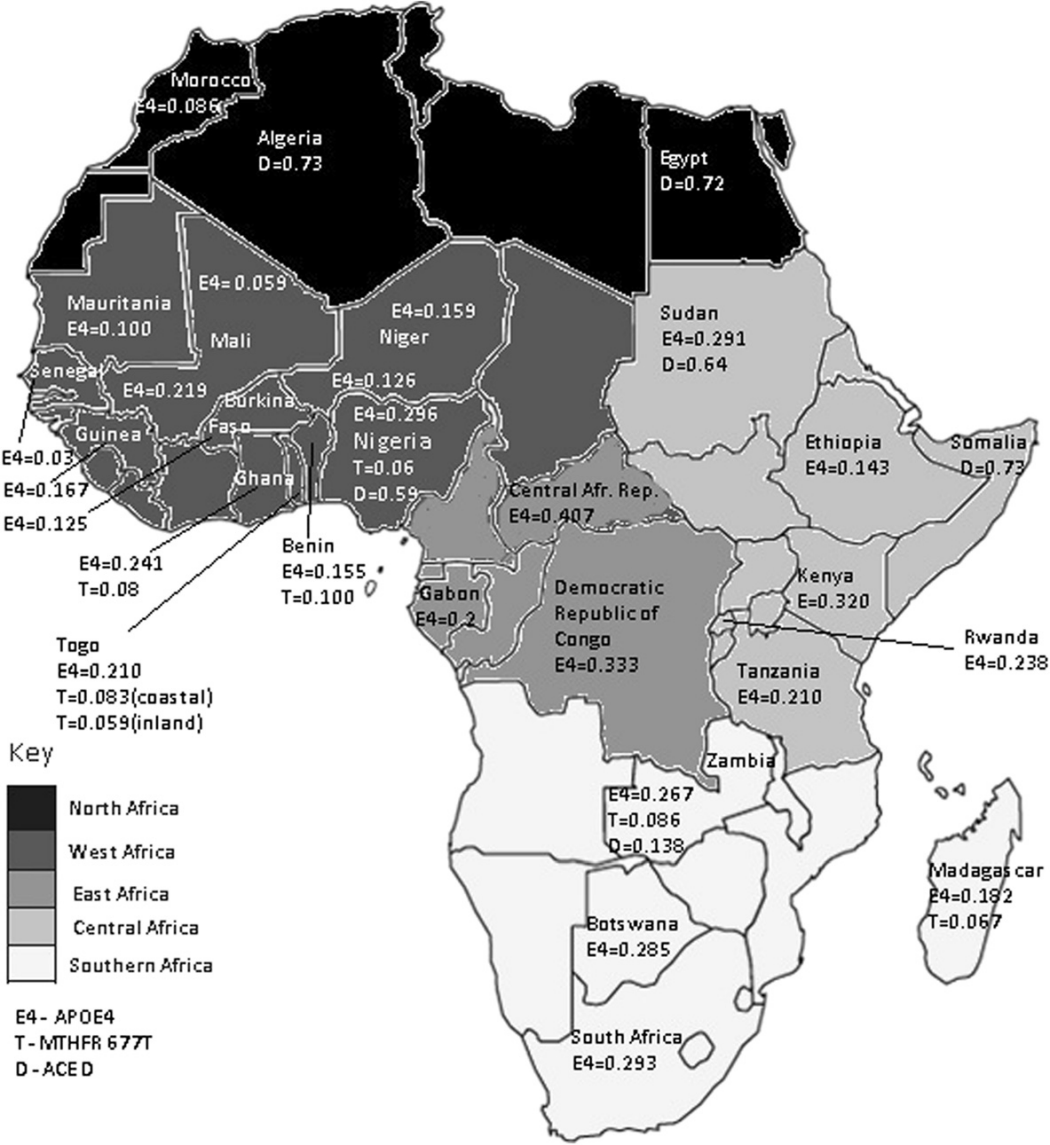
**Distribution of the APOE *ε*** **4, MTHFR 677 T and ACE D allele frequencies in the African populations.**

The frequency of *APOE* polymorphisms is highly heterogeneous among African [2,47,63] and other populations [26] (Figures 1,2). This heterogeneity illustrates the geographical and ethnic variations resulting from the evolutionary process. It has been suggested that *APOE* ε2 and ε3 alleles arose by mutation of the ε4 allele [19]. Past studies have showed that the ε3 allele is more widely distributed probably because of positive selection for this allele in these regions [19,26].

It was found that the *APOE* ε4 (ancestral) allele frequency is higher in the Khoisan population than the rest of the Bantu populations [2,26]. According to this data it was suggested that there is a flow of the ε4 allele from the Khoisan to other Bantu populations [19]. However, it is not clear if the increasing of CAD, CVD and AD in the African populations is associated with the ε4 allele frequency. Since the African populations are characterized by greater levels of genetic diversity [63], the association of CAD, CVD, and AD with the ε4 allele frequency needs to be investigated in each of the major ethnic groups of the Zambian population.

Our study showed that C allele of the *MTHFR* C677T polymorphism was higher than T allele (*p* < 0.0001) with the TT genotype completely absent. The frequency of *MTHFR* C677T polymorphism has not been widely studied in African countries. However, few published studies showed that the 677TT genotype and the 677 T allele are markedly lower (below 10%) in the African populations than other ethnic groups [40-46,65-68].

For instance whites in Europe had 677TT frequency range of 8% to 18% [40] while this frequency for whites outside Europe ranged from 10 to 14% [67]. Amerindians from Brazil had 677TT frequency range of 21% [40].

The difference in the *MTHFR* alleles among African and other ethnic groups is not yet known. Studies in last decade have shown positive correlation between concentration of folate and the frequency of the 677 T allele [43,45], and hypothesized of gene-nutrient interaction between MTHFR and folate status [43]. On the basis of these data it was suggested that adequate folic intake in a given population may increase the frequency of the 677 T allele as described in Europeans [45] and Americas [66]. Low frequency of the 677 T allele in African populations, including Zambian population, is probably influenced by folate deficiency due to malnutrition and infectious impairing intestinal absorption of folate [46,67].

It has been reported that in *P. falciparum malaria*, there is a correlation between plasma homocysteine (Hcy) concentrations and malaria severity [42,68]. Some authors suggested that the 677 T carriers with increased level of Hcy are more vulnerable to malaria in SSA [42]. Chronic malaria parasite infections in Zambia remain a significant risk factor for severe anemia, especially in children. Selection for the 677 T allele has been reported in relation to folate intake [69].

Unlike the *APOE* polymorphism, *ACE* I/D polymorphism has not been widely studied among the African populations. In this study, the frequency of the *D* allele in the Zambian population was significantly lower (*p* < 0.0001) than other African countries. This may be explained by important gene flow between the North African and other Eurasian populations, as it was suggested regarding *APOE* allele frequencies.

In our data, the *ACE* polymorphism frequency of the I allele was significantly prevalent compared to D allele (*p* < 0.0001). However, other studies have reported a high frequency of the *D* allele in the African populations [17]. The II genotype was higher than the ID genotype (*p* < 0.0001) and the DD genotype (*p* < 0.0001) in the Zambian population. The discrepancies in allele and genotype frequencies between Zambian and other populations need further investigations.

The frequency of the *D* allele of the *ACE* I/D polymorphism ranged from 0.03 among Australian aborigines to 1.00 among the Abazians in Europe [70-73]. It was reported that the DD was less frequent in Asians than non-Asians [73,74].

Previous investigations have suggested a genetic predisposition of *ACE* I/D polymorphism with CAD [75,76], CVD [77], diabetes mellitus [78] However, there were significant inter-ethnic variations [17,70,71]. There was no statistically significant gender difference in our study group for the investigated polymorphisms.

In interpreting the findings of this study it is essential to consider its limitations. The small sample size cannot be representative of all the Zambian population. However, this will be a stepping stone to larger ongoing clinical and genetic studies of stroke in the Zambian population. It is apparent that gender, age, ethnic tribe are important factors in the study for analysis of the candidate gene polymorphism for diseases. Other limitations of this study include lack of coverage from most African countries, particularly regarding the studies of the *MTHFR* C677T and the *ACE* I/D polymorphisms.

## Conclusion

We found that the *APOE* ε3 allele and the I allele of the *ACE* were significantly high in our study population while there were low frequencies observed for the *MTHFR* 677 T and *ACE* D alleles. Our analysis of the *APOE, MTHFR* and *ACE* polymorphisms may provide valuable insight into the understanding of the disease risk in the Zambian population. However, our findings need to be compared with the clinical implication through additional studies within the Zambian population. The present study will serve as a template for future investigations of the prevalence of these genetic markers in larger population samples and their possible association with CAD, CVD and AD in the Zambian population.

## Abbreviations

SSA: Sub-Saharan Africa; APOE: Apolipoprotein E; MTHFR: Methylenetetrahydrofolate reductase; ACE I/D: Angiotensin I-converting enzyme insertion/deletion; CVD: Cerebrovascular disease; CAD: Coronary artery disease; AD: Alzheimer’s disease; DNA: Deoxyribonucleic acid; HWE: Hardy-Weinberg equilibrium; MDS: Multidimensional Scaling.

## Competing interests

The authors declare that they have no competing interests.

## Authors’ contributions

MA has made substantial contribution to conception and design, drafted and revised the manuscript. MM has been involved in drafting and revising the manuscript, performed statistical analysis. PM participated in collection of data, participated in revising the manuscript. SL has been involved in revising the manuscript critically for important intellectual content. PBM has been involved in revising the manuscript critically for important intellectual content. SR participated in collecting of data. JFM has been involved in revising the manuscript critically for important intellectual content. OR has made substantial contribution to conception and design, carried out the molecular genetic studies, revised the manuscript, have given final approval of the version to be published. All authors read and approved the final manuscript.
